# Cheminformatics for the masses: a chance to increase educational opportunities for the next generation of cheminformaticians

**DOI:** 10.1186/1758-2946-5-32

**Published:** 2013-07-05

**Authors:** David J Wild

**Affiliations:** 1 Indiana University School of Informatics and Computing, Bloomington, IN 47401, USA

## 

As was observed in the opening editorial for this journal [1], cheminformatics is a discipline that has an unusually wide scope of application, from library science to molecular biology, yet has a small and scattered academic presence. There are many people keen to learn about the techniques of cheminformatics, but only a small subset of them are able to obtain a formal qualification in it, due to time and commitment constraints, financial limitations, and proximity to one of the sites offering degrees in the field. The programs available and some of these challenges have been reviewed previously [2,3].

Online learning is thus an enticing opportunity for cheminformatics. If done well, it could widely broaden participation in cheminformatics learning, removing most if not all of these constraints on learning, and enabling the wisdom gained over the long history of the field to impact a wide range of practitioners and disciplines. It could help draw together expertise from rapidly growing cheminformatics communities in India, China, Europe, North America, and elsewhere. Indiana University has been developing and experimenting with a variety of online techniques since the inception of the cheminformatics program there a decade ago, including offering IU classes for credit synchronously to remote students through video and web conferencing [4], free online learning resources for cheminformatics [5], and a low cost online introductory cheminformatics textbook in PDF format [6]. A variety of other resources have been made available online, of particular note the *Henry Stewart Talks* Introduction to Cheminformatics series [7].

We are currently in the midst of a revolution, or at least a potential revolution, in how learning is done in higher education. *MOOCs* (Massive Open Online Courses), such as those offered by Coursera, edX, and Udacity, amongst others, offer the enticement of opening high quality education by world experts to anyone with an internet connection and computer. Universities are beginning to partner with these companies to offer accredited degrees, often at much lower cost than a traditional degree (see, for example, the recent announcement of an online M.S. in computer science by Georgia Tech and Udacity [8]). Much is still to be worked out in how to engage and retain students in online courses, avoid cheating, and so on, but it is clear that modern internet technologies are likely to vastly change the educational landscape, even if current offerings are just a transitional phase.

There are many ways in which this movement could widen the impact of cheminformatics on the world, and increase the number of people with expertise in its many techniques and applications. For example, I am collaborating with Robert Belford at the University of Arkansas at Little Rock, on a project to develop online MOOC-like cheminformatics teaching modules that can be used to create custom, locally organized cheminformatics courses for chemistry undergraduates at institutions that do not have the resources to teach cheminformatics themselves [9]. Localized expertise could be encapsulated in online resources and modules, and shared with Creative Commons licensing to allow customized training and accredited education options to be built for particular purposes in both academia and industry. The field as a whole could be advanced by online discussion around these modules (a Google + community exists precisely for this kind of discussion [10]).

In the opening editorial for this journal, we expressed strongly our belief that cheminformatics be centered and cemented as a true, distinct discipline, with a mechanism to educate the next generation of researchers. The ballooning importance of health, science, data and informatics in the 21st century is evidence of the critical importance cheminformatics will play in the coming years. At the *Journal of Cheminformatics* we therefore strongly support the broadening and increase of accessibility to cheminformatics education. We encourage those involved in cheminformatics education to submit papers to this journal on their experiences and vision as well as describing open resources that are being made available. We hope thus to spur a lively discussion on how to contextualize cheminformatics education in the globally connected world we live in. We further challenge those involved in chemistry and cheminformatics education to consider widening accessibility of materials, particularly through making materials available online.
